# Digging Into Dermatillomania: Scalp Reconstruction in a Complex Patient

**Published:** 2021-03-27

**Authors:** Sydni Meunier, Maelee Yang, Joseph Ogrodnik

**Affiliations:** Division of Plastic and Reconstructive Surgery, Department of Surgery, Loyola University Chicago Stritch School of Medicine, Maywood, IL

**Keywords:** dermatillomania, body-focused repetitive behavior (BFRB) disorders, full-thickness scalp wound reconstruction, dermal matrix templates, multidisciplinary treatment approach

## DESCRIPTION

**Figure 1:**
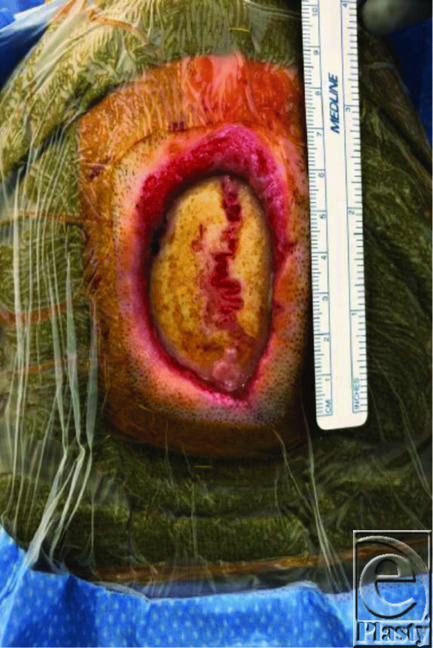
Preoperative photograph. Patient lying supine; right 6 x 4-cm paramedian oval defect.

**Figure 2:**
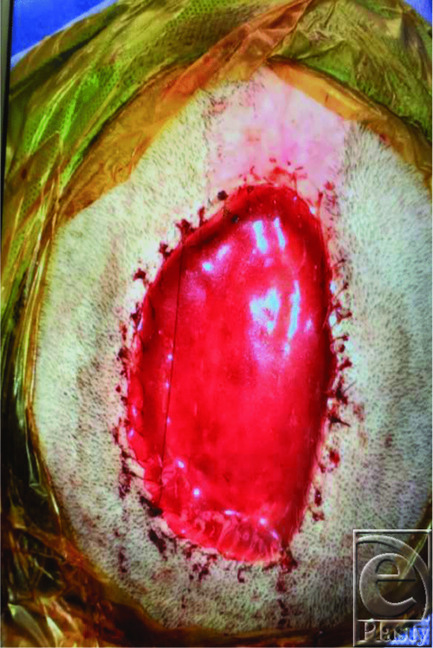
Surgical photograph. Taken after neurosurgery assessment, plastic and reconstructive surgery debridement, and Integra placement and securement.

**Figure 3:**
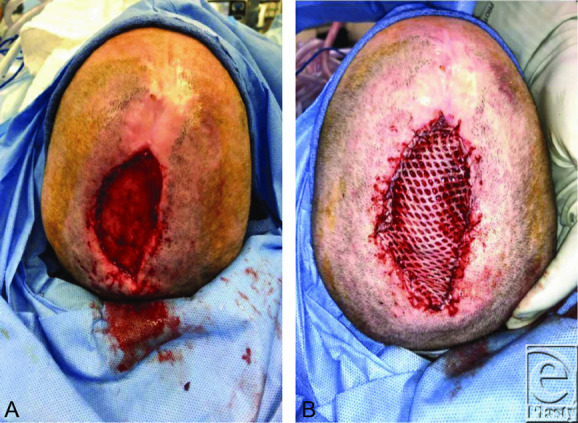
Surgical photograph. Five weeks post-Integra placement. (*a*) Integra silicone outer layer removed with evidence of graft vascular integrity. (*b*) After placement of meshed split-thickness skin graft.

**Figure 4:**
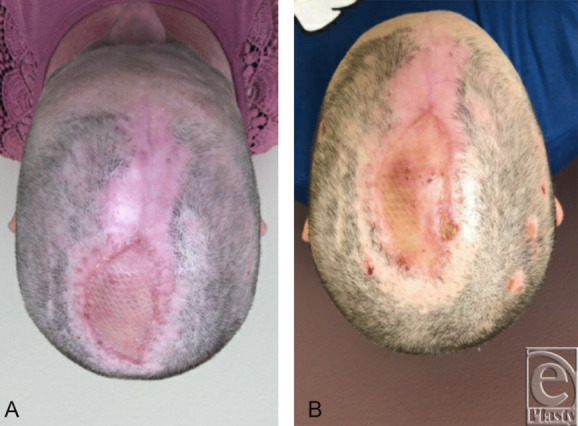
Postoperative photograph. (*a*) Three-week follow-up from split-thickness skin graft placement. (*b*) Three-month follow-up.

C.W. was a 39-year-old white woman, mother of 5, with a history of depression who presented with an enlarging full-thickness right paramedial scalp wound (Fig 1). Over the past year, the patient was noted by multiple family members to be compulsively picking her scalp until she finally presented by the urging of her mother with exposed calvarium. Social history was pertinent for mothering 5 children along with multiple pets at home including cats and a bearded dragon. Psychiatry, neurosurgery, and plastic and reconstructive surgery (PRS) were consulted. Her depression had been worsening, but she had not sought out any additional treatment. Psychiatry diagnosed her with body-focused repetitive behavior (BFRB) disorder, starting her with fluoxetine and cognitive behavioral therapy (CBT). Neurosurgery denied indication to intervene, as the calvarium was intact and there were no neurological deficits. PRS then performed a 2-stage reconstruction of the 6 × 8-cm defect. Stage 1 included debridement, bone burring and placement of a dermal matrix template, and negative pressure wound therapy (NPWT) (Fig 2). Stage 2 took place 5 weeks later with placement of a meshed split-thickness skin graft (Fig 3). Reconstruction was successful, and the patient is doing well amidst ongoing psychiatric challenges (Fig 4).

## QUESTIONS

1. What is dermatillomania and BFRBs and how are they treated?
2. What are the adverse consequences of large full-thickness scalp wounds, and why are they difficult to repair?
3. How are dermal matrix templates useful in full-thickness scalp wound reconstruction in the setting of dermatillomania?
4. What kind of team is necessary for successful scalp wound reconstruction in dermatillomania?

## DISCUSSION

BFRBs are disorders that fall under obsessive-compulsive and related disorders according to the *Diagnostic and Statistical Manual of Mental Disorders* (Fifth Edition) and the American Psychiatric Association.^1^ BFRBs include dermatillomania (also called skin excoriation disorder) and trichotillomania, skin and hair picking, respectively. These disorders are relatively common, estimated to be found in 2% to 5.4% of the population, up to 12% in some studies, and most frequently target the face and scalp.^1^^-^5 These diagnoses are distinct from self-mutilation disorders as they are not with intent to harm; rather, they are performed with varying degrees of consciousness. They often accompany anxiety or boredom or result in gratification, pleasure, or relief when performed. BFRBs are often comorbid with other psychiatric illness, most significantly major depressive disorder, anxiety, and obsessive-compulsive disorders.^4^^,^^5^ Mrs C.W., a female presenting with scalp picking and comorbid depression, represents the most inflicted demographic as described in the literature.

BFRB defects span widely beyond suboptimal aesthetics and can be significantly dangerous or permanent. Scarring, localized infection, systemic infection, osteomyelitis, and anemia secondary to blood loss have been reported.^4^^,^^5^ When located on the scalp, there is further concern for brain matter injury, epidural abscess, seizure, and neurological deficits. With these consequences, quick and effective treatment is necessary. Full-thickness scalp reconstruction has been discussed prevalently in the literature with optimal outcomes. Research has examined techniques including primary closure, tissue expanders, flaps, free grafts, and allografts and how each address the surgical complexities of scalp reconstruction such as limited tissue laxity, complex vascular supply, and alopecia. In this patient even before consideration of her psychiatric diagnosis in the surgical planning, the location of the defect as well as its large 48-cm^2^ size limited potential success by primary closure or tissue expanders. Grafting was foreseen to have similar unfavorable outcomes due to the absence of periosteum, leaving an unsuitable wound bed with poor vascularity.^6^^,^^7^

Dermatillomania demanded further individualized consideration in surgical planning. Continued picking behavior carried concerns for acute worsening of her clinical status by any one or multiple of the aforementioned complications. Probable success necessitated a technique providing timely, complete debridement and full coverage of the wound bed. In traumas, burns, and oncology, dermal matrix templates have been successful on large reconstructions.^7^^,^^8^ Dermal matrix template on burred calvarium also allows rapid and complete closure after a single procedure and is successful despite compromised vascularity. An allograft (in our case, acellular Integra derived from shark cartilage) circumvents the need to locate a viable, unpicked flap donor site. This prevents creation of a second defect that might provoke patient picking behavior. While there was eventual need for a donor site for the second-phase split-thickness skin graft, these harvests are generally less involved and performed later, allowing more lapse for psychiatric care. The choice of NPWT for wound dressing added additional insurance to the success of the procedure. The sealed, bandaged apparatus provided a short-term protective barrier from skin picking in addition to its propensity for improved wound healing.

Despite a relatively high prevalence of BFRBs, there is a lack of screening, diagnosis, and subsequent report on how to treat them and their complications. This is problematic, as these disorders necessitate unique treatment considerations in order to achieve successful outcomes, as in our patient. Our compilation and utilization of a multidisciplinary team involved the expertise of psychiatry, neurosurgery, and PRS. With the concerns of each, a cumulative plan and realistic timeline could be made, accounting for the intricate, dependent comorbidities and maximizing the use of personnel and resources. Psychiatric treatment as the first intervention avoided misspent surgical efforts and materials that would be seen in a patient who quickly resumes picking behavior at the site, as well as alerted the team to a patient who would need long-term, close follow-up to ensure patient compliance. Assessment of neurological status avoided reoperation to explore an underlying problem, potentially missed in haste to close the wound. PRS surgical technique could then be devised to maximize speed and success of an already challenging reconstruction but minimize provocation of picking behavior. At PRS follow-ups, the importance of continued psychiatric care could be reiterated and emphasized.

BFRB-inflicted scalp wounds present a complex surgical reconstruction complicated by the threat of self-picking behavior. With the prevalence and chronic nature of BFRBs, we predict that reconstruction of inflicted defects is largely underrepresented in the literature. We have demonstrated a successful multidisciplinary approach to this comorbidity, utilizing early and continued psychiatric intervention to maximize outcomes of timely PRS allografting of full-thickness scalp wounds.
